# Single-cell sequencing in molecular diagnostics: Transformative yet untapped potential

**DOI:** 10.3389/fgene.2025.1621081

**Published:** 2025-11-19

**Authors:** Duaa Elshiekh, Nour Farchoukh, Reema El Hassan, Sara Al-Marzooqi, Waad Rashed Aldosari, Haissam Abou-Saleh, Amal Al-Haidose, Hatem Zayed, Atiyeh M. Abdallah

**Affiliations:** 1 Department of Biomedical Sciences, College of Health Sciences, QU Health, Qatar University, Doha, Qatar; 2 Forensic Laboratory Department, Ministry of Interior, Doha, Qatar

**Keywords:** single-cell sequencing, clinical molecular diagnostics, personalized treatment, data analysis challenges, omics

## Abstract

Single-cell sequencing (SCS) techniques have the potential to offer precise insights into cellular diversity by revealing unique genetic and transcriptomic profiles at the single-cell level. This advanced technology has been used extensively in research, but it has still not translated to clinical diagnostics, despite its potential. SCS provides more accurate granular information about heterogeneous cell populations and the creation of personalized treatment strategies. However, the integration of SCS into clinical practice is challenging. This review discusses the potential of SCS technologies in improving clinical molecular diagnostics in various clinical areas including oncology, genetics and rare diseases, infectious diseases, and autoimmune disorders and inflammation. We review recent advances, current uses, integration challenges, and their contribution to the development of these fields. SCS provides significant opportunities in oncology to analyze tumor heterogeneity and develop personalized treatments. In autoimmune and rare diseases, SCS has helped to define novel biomarkers and understand complex immune pathways. SCS has also been used to understand pathogen diversity and host-pathogen interactions in the context of infectious diseases, leading to targeted therapeutic approaches. Despite this progress, there remain challenges in data analysis, standardization, and routine clinical application. The future of SCS in clinical molecular diagnostics is promising. Further technological and research developments in SCS are expected to increase the precision and personalization of medical diagnostics and treatment. To overcome current limitations, interdisciplinary cooperation and innovative approaches to data analysis are needed.

## 1 Introduction

**FIGURE 1:**
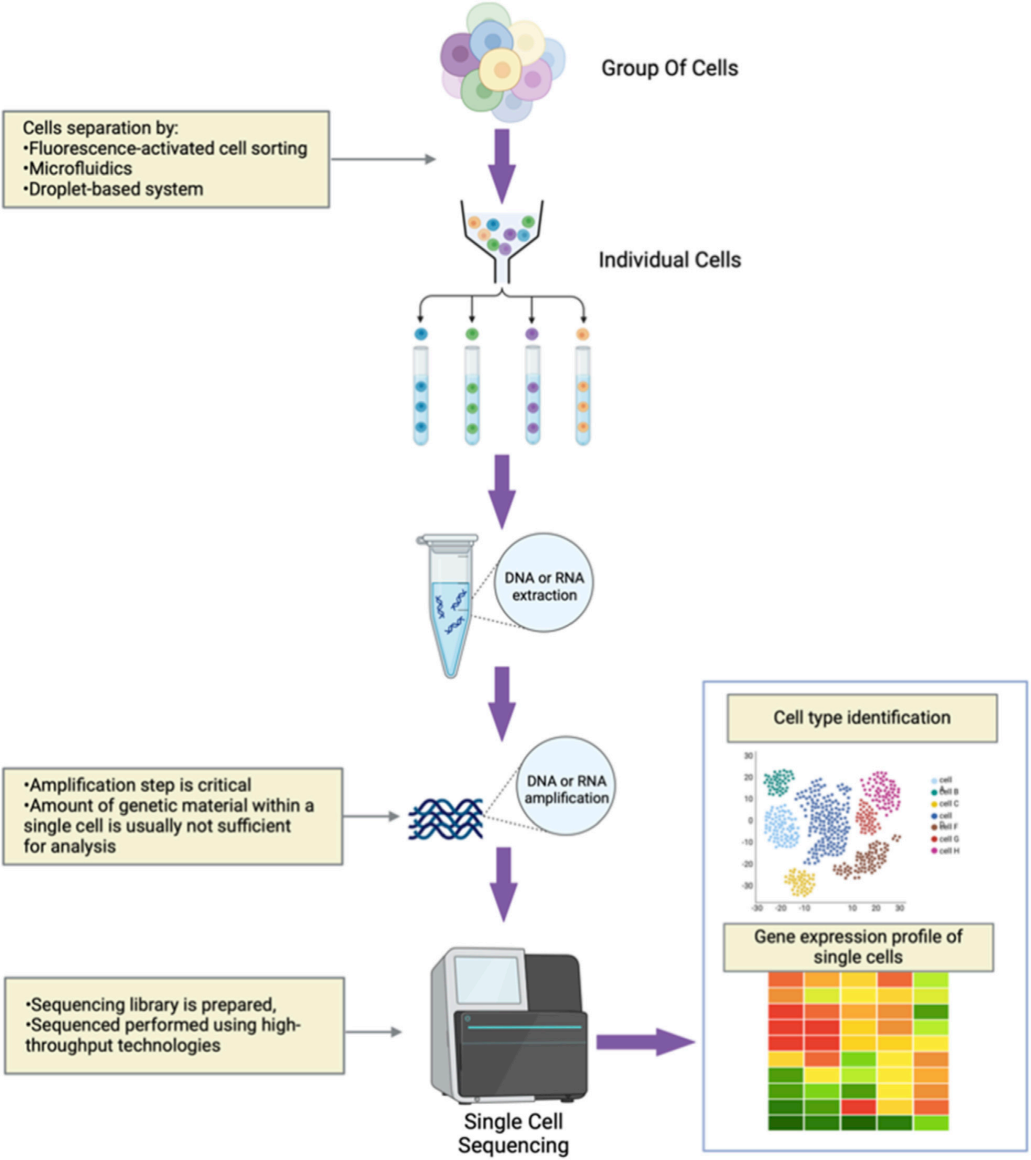
An overview of single-cell sequencing (SCS). Groups of cells isolated from a sample are separated to produce individual cells. Then, nucleic acid is extracted and amplified from single cells for sequencing and further analysis.

Conventional sequencing techniques average the signal from thousands or even millions of cells in an individual sample. By contrast, single-cell sequencing (SCS) is an advanced, cutting-edge technology that enables the sequencing and analysis of individual cellular genomes, transcriptomes, and epigenomes (Wen and Tang, 2022). In this way, SCS can provide a detailed understanding of the genetic and functional variations at the cellular level (Yu et al., 2021). SCS is a multi-step process that first uses techniques such as fluorescence-activated cell sorting (FACS), microfluidics, or droplet-based systems to isolate single cells from a tissue sample for analysis. DNA or RNA is then extracted from individual cells and amplified, with this latter step critical for providing sufficient genetic material for analysis. Then, a library is prepared for sequencing using high-throughput technologies. The resulting data are analyzed to annotate distinct cell types and their genetic profiles (Figure 1) (Clark et al., 2020; Mazutis et al., 2013).

Since its introduction, SCS has provided unprecedented detail of the characteristics of cells within heterogeneous tissue populations (Choi and Kim, 2019). The high specificity and sensitivity of SCS make it a promising technique for clinical research and applications, including molecular diagnostics (Zou D. et al., 2021). Cellular heterogeneity is a critical pathophysiological factor in many disease states including cancers, infections, autoimmune diseases, and complex and rare genetic diseases (Huang W. et al., 2021; Jia et al., 2022; Mi et al., 2021; Montgomery et al., 2022). SCS provides detailed insights into the complexity of disease development and reveals possible new mechanisms of disease initiation and progression (Han et al., 2022). Additionally, SCS can contribute to the discovery and development of novel biomarkers for clinical diagnostics, prognostics, and drug response prediction, which may be more sensitive and reliable as they are based on detailed information of single cell alterations. Additionally, SCS has contributed to drug discovery through its ability to more accurately identify and assess cellular responses to targeted therapies (Zou D. et al., 2021; Sultana et al., 2023). By fully embracing cellular complexity, SCS is paving the way for a new era of personalized medicine and enhanced individualized patient care (Towle-Miller and Miecznikowski, 2022).

SCS and bulk sequencing are both commonly used in genomics, but the data generated from these techniques differ widely in both detail and granularity (Li and Wang, 2021). Thus, it is important to understand and differentiate the most appropriate applications for each technique. The choice of method depends on the specific research objective, the anticipated data resolution, and, of course, budget constraints. While bulk sequencing is currently less expensive than SCS for obtaining a broad comprehensive view of expression profiles, SCS offers detailed insights into the expression profiles of individual cells and tissue heterogeneity at high sensitivity, specificity, and resolution, as shown in Table 1 (Kuksin et al., 2021; Li et al., 2021).

**TABLE 1:** A comparison of SCS and bulk sequencing.

Type of sequencing	Resolution and sensitivity	Data interpretation	Cost and complexity	Insight into diseases	Uses
Bulk sequencing	-Average view of genetic expression -Masks contribution of rare cell types	Identifies common themes and signals	Generally, less expensive and less complex	-Identifies dominant genetic alterations -Provides an overview of changes in the genetic landscape over time	-Used when the aim is to identify overall gene expression patterns -When sample heterogeneity is low
SCS	-Detailed view of individual cells gene expression -Allows detection of rare cell populations	Identifies cellular diversity and dynamics	More expensive and technically demanding	-Identifies rare genetic alterations -Can monitor clonal evolution of new subpopulations	-Used when the aim is to understand cell-cell variations -Useful when sample heterogeneity is high

SCS could therefore revolutionize clinical molecular diagnostics by providing an unprecedented level of detail and precision to positively impact the diagnosis and treatment of a wide range of diseases, especially those where cellular and subcellular heterogeneity drive the pathogenesis of disease and responses to therapies. In this review, we evaluate the current landscape, recent advances, barriers, and contributions of SCS to oncology, genetics and rare diseases, infectious diseases, and autoimmune disorders and inflammation.

## 2 Fundamental principles of single-cell sequencing technology

SCS describes the sequencing of single-cell genomes (DNA) or transcriptomes (RNA) to obtain multi-omics (genomic, transcriptomic, and epigenomic) data in a high-throughput and cost-effective manner (Pensold et al., 2020). Classical sequencing methods are applicable only to groups of cells, and the approach averages cellular heterogeneity. However, SCS analyzes small numbers or individual cells, detects heterogeneity, and generates cell maps for clinical research to facilitate personalized medicine (Tang et al., 2019). SCS has grown in popularity over the last 7 years and is now used in every biomedical discipline including oncology, immunology, neurology, and microbiology (Proserpio et al., 2022).

### 2.1 Cell sorting

SCS is a sequential process that first requires cell isolation and preparation, the most relevant being FACS (fluorescent activating cell sorting), microfluids, LCM (laser capture microdissection), or manual cell picking (Gross et al., 2015). The preparation and isolation of single cells can be challenging in terms of obtaining sufficient yield, sensitivity, throughput, and quality. The method used depends on the nature and origin of the sample.

FACS uses laser excitement to generate metrics on cellular properties like size and granularity (forward or side scatter) and, if stained with florescent markers, also a readout of functional properties. A cell stream is formed using nozzles to form the hydrodynamic jet and, through targeted vibrational actuation, the jet breaks to collect cells of interest by droplet deflection using electrical plates that guide cells into small tubes or microwells (Zeb et al., 2019).

LCM is an advanced tool used to isolate cells from solid samples (i.e., tissues) using lasers to precisely dissect the sample. The tissue is visualized by microscopy, and the area of interest for cutting is drawn for subsequent extraction via three methods: contact-based extraction, contact-free gravity-assisted microdissection (GAM), and contact-free laser pressure-capturing (LPC) (Hu et al., 2016). In contact-based extraction, after laser cutting, an adhesive cap/tube is used to extract the released cut segment. In GAM, the sample is placed over a collection tube such that the cut section falls into the receptacle using gravity.

Microfluidics achieves single cell separation using lab-on-chips, with popular devices using droplet in oil-based, pneumatic membrane valving, and hydrodynamic cell traps (Pensold et al., 2020). In droplet in oil-based systems, each droplet in oil-filled channels contains a single cell, and the system holds the aqueous droplet in a sieve or emulsion. Isolation of single cells is either random or with 80% yield. One major advantage of this approach is its high throughput and capacity to process thousands of cells per second (Gross et al., 2015). Pneumatic membrane valving employs pressurized air to deflect an elastomer membrane to close the microfluidic channel, but the approach is low throughput (Briones et al., 2021). Hydrodynamic cell traps use passive elements to hold single cells in position. This method is designed to reduce double occupation via adjustment of the trap size to the average cell size (Deng et al., 2020).

Finally, magnetic-activated cell sorting (MACS) is also used to generate cost-effective, high-throughput, high purity, and targeted separation of cells for SCS. In MACS, magnetic beads are used to separate cells using specific markers (usually antibodies) to ensure efficient and specific purification and enrichment. MACS achieves up to 98% purity of stem and immune cells (Korkusuz et al., 2019). One major advantage of MACS is the ability to perform both positive and negative selection (Hu et al., 2016).

### 2.2 Sequencing technologies

As sequencing technologies evolved, Illumina provided embedded SCS in next-generation sequencing (NGS) technologies, such as with the NextSeq1000/2000 systems and NovaSeq X Series. These technologies are highly sensitive with a low-input workflow to enhance heterogeneity and functional studies in time-dependent assays such as proliferation, differentiation, and tumorigenesis. The workflow starts with primary tissue preparation, single cell isolation and library prep, sequencing, and primary analysis followed by data visualization and interpretation. Tissue preparation is highly dependent on the cell type and microenvironment to provide viable cell suspensions through mechanical isolation, enzymatic digestion, or their combination. Microfluidics and microwells are mainly used for single-cell separation, after which they are lysed for target library preparation and then each target is barcoded. Targets of interest are selected for amplification and prepared for sequencing by loading the library into an appropriate sequencer. Generated data are stored and shared in the BaseSpace sequence Hub, which allows for further data analysis and processing. New SCS approaches developed by Illumina focus on quality control for SCS experiments, multiplex sequencing, and combining different technologies (Wu et al., 2014). Data are analyzed and interpreted with the DARGEN Bio-IT platform, which uses a cell-by-gene expression matrix output pipeline for downstream analysis. Key features include rapid analysis (<40 min) for ∼8000 cells, generating >1 billion reads, high compatibility with a range of library types, and efficiency (Illumina, 2014).

A comparative study of the NextSeq 500, NovaSeq 6,000, and BGI MGISEQ-200 (Wu et al., 2014) platforms revealed that the NovaSeq 6,000 and MGISEQ-200 were similar in terms of analytic outcomes and general analysis, although Illumina sequencers exhibited advanced cell barcoding for base calls compared with the BGI MGISEQ-200.

High accuracy reads were first provided using short-read sequencing. Although short-read sequencing revolutionized biomedical research, the technology misses around 80% of the transcripts due to 3′ or 5′ bias. More recent third-generation long-read sequencing, such as that provided by Oxford Nanopore Technology (ONT), enables the generation of long reads for genomics and transcriptomics (10 Mb and 10 kb) at single-cell level. The ONT MinION, GridION, and PromethION sequencers measure ionic current fluctuation when single strands are passed through the pore, which facilitates base calling. Each nucleotide produces a specific current variation reflecting the resistance to stretch in the pore (Hayrabedyan et al., 2021). EPI2ME labs also provides a collection of next flow bioinformatics that supports long-read sequencing analysis. One advantage of this analytical tool is its versatility for different workflows based on the field of interest (human genetics, assembly, metagenomics, direct RNA sequencing and cDNA, infectious diseases, targeted sequencing, and miscellaneous). The approach not only identifies single cells by base calling but also implements unique molecular identifiers (UMIs) for barcoding to ensure error correction and to increase accuracy.

Given the wide variety of available technologies, the main barrier to clinical utility is a lack of protocols to integrate such data into biomedical and translational research. Data integration is relevant when measuring specific modalities within a single cell (Stuart et al., 2019). Secondly, meta-data could improve analyses for future experiments (Haque et al., 2017).

All the techniques discussed above provide insights into cellular heterogeneity and function. Quality control (QC) is essential for achieving sequencing objectives. A newly developed pipeline, *singleCellTK* package (SCTK-QC), is a standalone script that can be incorporated into cloud-based pre-processing pipelines for QC. The approach not only maintains the quality of sequencing but facilitates data import and cross-compatibility to establish comprehensive sets of QC metrics. The main concept is to overcome challenges inherent in scRNA-seq data, as current tools are implemented in different programming environments. Users must download and install each tool independently for each sample, which is time-consuming. Furthermore, a lack of standardized workflows for QC metric generation is a barrier to converting sequencing outputs into meaningful translation of such data into diagnostic applications (Tang et al., 2019; Hong et al., 2022).

### 2.3 Analytical tools

SCS analysis tools, algorithms, data filtering, clustering, and differential expression analysis are evolving. Traditional pre-processing and clustering methods have limitations that prevent data analysis in different cell or tissue microenvironments, including high dimensionality, noise, sparsity, and large-scale characteristics (Wang et al., 2022). There is now a move towards using deep-learning models to take important factors such as data processing into account, including QC, data correction, normalization, visualization, dimensionality reduction, and cell clustering for downstream analysis. A pivotal issue in SCS analysis is data generalization, as most machines analyze cells as batches, introducing batch effect, especially in tissue-based studies. Recent solutions focus on the ability to either reduce or completely remove this effect, as it can eliminate heterogeneity information for less abundant cells. Batch effect should ideally be removed during analysis to allow data normalization, but it is fundamental to data clustering. Solutions should consider a system that establishes balance between the on/off batch effect. Overall, batch effect takes two forms: embedding space, where cells are sequenced individually, and gene expression space, where cell grouping is needed (Lakkis et al., 2021).

Gene variability can occur due to several internal or external factors. Internal factors include biological causes, while external factors include technical differences, namely, noise and missing values as a result of (false zero) or dropout events. Zeros may be true zeros reflecting no gene expression in the cell or false zeros due to low capture rates or unequal sequencing coverage. Denoising or imputation have been proposed to address this problem through detection of dropout events. Although these methods highlight the importance of data correction, true data can be altered through overcorrection. This problem increases demand for more controllable tools, such as the scImpute system, which estimates dropout probability among genes and addresses those with higher values, after which false zero values are corrected to preserve cellular heterogeneity for true 0 cells. There is still no perfect bioinformatic method to detangle noise and missing values in sequencing data. Available approaches tend to either provide excessive or insufficient imputation, so tool selection should be based on the data characteristics and research objectives.

Cell clustering relies on the accurate identification of cell types. Cells are clustered based on gene expression similarity. Three main algorithms are used for clustering: K-means, hierarchical, and consensus clustering. However, these approaches may generate clusters of equal sizes missing rare cell types. They can also be time and memory expensive, making them inappropriate for large datasets. To solve these problems, new methods reduce the dimension of the analysis to permit data integration, such as with scDeepcluster, scSemicluster, and scCCESS models. scDeepcluster implements an autoencoder that uses two functions, the first to generate low-dimensional representations and the second to transform noise to denoise data and fill in missing values. scSemicluster employs target and reference data for training, testing both discriminative and generative factors of the target, resulting in structure-based similarity. scCCESS integrates an autoencoder artificial network that first uses random sampling N times, and then each sub-dataset generates N numbers followed by individual mapping at low-dimensional testing (Wang et al., 2022).

Differential expression (DE) analysis is used to determine differences in the expression of genes under different conditions. Conventional DE data are heterogenous, with high zero counts (sparse data), though improvements in SC analytics have been directed towards average non-zero DE calculation. A key issue with sparse data is that scRNA-seq often captures only a fraction of a cell’s RNA molecules, leading to many zero values in the expression matrix. These “dropouts” can mask true biological signals and inaccuracy in DE results. scRNA-seq experiments often use a smaller number of biological replicates than bulk RNA-seq experiments, which reduces statistical power to detect subtle changes in gene expression. To analyze sparse data, normalization methods like scaling and size factors are used to account for technical variations, while imputation techniques aim to fill in missing values (zeroes) with statistically estimated values. Popular imputation methods include KNN and matrix factorization approaches like WEDGE (Zhang M. et al., 2022). An alternative solution is to use specialized DE tools, as traditional DE tools designed for bulk RNA-seq data often struggle with sparse data. Newer tools like DESeq2, edgeR, and scDEseq are specifically designed for scRNA-seq data and account for the inherent dropout rates and noise. These tools use statistical models that consider the negative binomial or Poisson distribution for read counts, which are better suited for modelling over dispersed count data with many zeros. Finally, alternative approaches like clustering analysis can be used to identify groups of cells with similar expression profiles, followed by DE analysis between clusters (Alessandrì et al., 2019). This can be particularly useful for identifying rare cell populations not readily apparent in standard DE analysis. The optimal technique to use depends on several factors including the specific research question, the type of scRNA-seq data (e.g., droplet-based vs. plate-based), and the desired level of sensitivity and specificity (Nguyen et al., 2023). Results from different tools should be compared and their performance assessed for a particular data set.

## 3 Clinical applications of single-cell sequencing

### 3.1 Oncology

Cancer biology - and applying this knowledge to the clinical discipline of oncology - are complex. Malignant tumors are characterized by a high degree of heterogeneity that not only challenges understanding tumor biology but also complicates the diagnosis and development of effective treatments (Pe’er et al., 2021; Zhang A. et al., 2022). Tumor heterogeneity describes the presence of genetically, epigenetically, and phenotypically distinct subpopulations of cancer cells in the same tumor, in tumors at different sites in the body (such as between primary and metastatic sites), or in different patients. Tumor heterogeneity can therefore be intratumoral (diversity between cancer cells within the same tumor) or intertumoral (diversity between tumors in different patients or different tumors in the same patient) (Danilenko et al., 2021). It arises from the accumulation of somatic mutations, copy-number changes, and epigenetic modifications, as well as through the actions of the tumor microenvironment such as hypoxia, immune pressure, and stromal interactions (Proietto et al., 2023). Driver mutations convey a selective growth advantage and increase tumor growth, while so-called passenger mutations arise without functional consequences (Proietto et al., 2023). While bulk sequencing could not adequately address tumor heterogeneity and complexity (especially for intratumoral heterogeneity) (Nath and Bild, 2021), SCS provides the resolution to significantly advance oncology by quantifying and understanding tumor heterogeneity through comprehensive mapping of the genetic and phenotypic variabilities of tumors on a cell-by-cell basis. The expectation is that SCS will eventually lead to the development of enhanced diagnostic strategies and treatments for individualized oncology care.

#### 3.1.1 Single-cell resolution of tumor heterogeneity

Cancer is a dynamic disease where cells increase in diversity as the disease progresses. The high level of intratumoral heterogeneity means that cells within the same tumor can exhibit different cellular morphology, phenotypic profile, gene expression, metabolism, and metastatic potential. These differences complicate not only our understanding of cancer but also how we deal with it (Egeblad et al., 2010) as, in the clinical setting, identifying distinct tumor subpopulations is critical, as these differences contribute to metastasis, disease progression, drug resistance, and responses to treatments (Huang RH. et al., 2021; Maleki et al., 2024). SCS is a powerful tool to identify different cell populations within tumors as well as rare tumor cells with unique mutations or phenotypes. For instance, SCS makes it possible to identify cancer stem cells, which are implicated in metastasis and therapy resistance but are only present in small numbers in tumors or circulating in the bloodstream (Chu et al., 2024). These are critical to identify so that their unique, cancer-promoting characteristics can be targeted therapeutically (Frąszczak and Barczyński, 2024). SCS enables the identification of these cells, tracks their evolution, and enables the development of targeted therapies to prevent metastasis and improve patient outcomes (Mannarapu et al., 2021; Xu et al., 2021). As a result, it is critical to identify the different subpopulations within the tumor to tailor more efficient treatment approaches.

SCS also has value in analyzing the expression profiles of the other (non-cancer) cells in the tumor microenvironment. For example, in a 2020 study, SCS identified prognostic microenvironment marker genes and signatures in single-cell profiles of lung adenocarcinoma, paving the way for targeting these profiles for precision medicine (Bischoff et al., 2021). Cancer heterogeneity also arises from epigenetic modifications (Sadida et al., 2024), which are significant regulators of gene expression that contribute to the initiation and spread of tumors, and SCS can also be utilized for single-cell epigenomic profiling (e.g., DNA methylation and histone modification landscapes). These epigenetic modifications can be targeted for cancer diagnosis, prognostication, and treatment (Hu et al., 2023).

#### 3.1.2 Dynamic monitoring of tumor evolution and resistance mechanisms

SCS can also be used to track tumor evolution and resistance mechanisms over time to provide insights into the intricate behavior of cancers during their growth, metastasis, and in response to treatment and immune responses (Kuipers et al., 2017; Mossner et al., 2021). For example, SCS was used to study patients with triple-negative breast cancer who had received neoadjuvant chemotherapy, which revealed resistant, pre-existing subclones that were adaptively selected. However, their transcription profile underwent reprogramming in response to the chemotherapy, suggesting a combined intrinsic and acquired model of drug resistance (Kim et al., 2018). SCS can be used to monitor evolutionary changes over time, and identifying these evolutionary alterations in tumor subclones could reveal potential cancer vulnerabilities for specific targeted therapies.

Even more recently, combining SCS with spatial transcriptomics has revealed the genetic variability of cancer cells across different regions of the tumor and at sites of metastasis. This approach can be used to understand the genetic and functional changes occurring in tumor cells located at metastatic sites and comparing them to the original tumor, such that the metastatic subtypes can be targeted therapeutically (Ahmed et al., 2022; Zhu and Qin, 2023). Moreover, visualizing the cells enables identification of clonal expansion of resistant tumor subpopulations, allowing timely adjustment of treatment strategies to target emerging resistant populations (Liu et al., 2021; Ren et al., 2022). For example, a study of patients with cervical cancer showcased the utility of SCS to identify mechanisms of resistance. By studying differential expression profiles of chemo-sensitive and chemo-resistant patients, the study revealed that the PI3K/AKT pathway was involved in progression of cervical cancer and resistance to therapy (Gu et al., 2021).

#### 3.1.3 Identification of novel biomarkers for early detection and predicting treatment responses

SCS can significantly enhance the utility of liquid biopsies and non-invasive monitoring by analyzing circulating cancer cells in body fluids such as blood (Adhit et al., 2023). The high resolution of SCS allows the detection of low abundance cells and fragments, which was not possible with bulk sequencing. The non-invasive nature of liquid biopsies and the detailed insights offered by SCS make it possible to monitor cancer progression or treatment responses easily without the need for invasive procedures (Kim et al., 2021). Additionally, this method can be used to identify novel biomarkers or test for the presence of known biomarkers (Li et al., 2018; Shen et al., 2024). By analyzing blood samples at various stages of the disease or in response to treatments, it may be possible to identify the disease state, therapeutic efficacy, or potential relapses. Since it is minimally invasive, blood sampling can be conducted frequently over the course of treatment, facilitating continuous accurate monitoring of disease progression (Kidess and Jeffrey, 2013). This allows for the identification of novel biomarkers for diagnostics, prognostics, and streamlining therapy selection and prioritizing treatments most likely to be effective (Peneder et al., 2021). For example, SCS was performed on patients with non-small cell lung cancer (NSCLC), which identified 12 key genes including *MS4A1*, *CCL5*, and *GZMB* as potential diagnostic and prognostic biomarkers (Sultana et al., 2023). In another study investigating human lung cancer transcriptomes at the single-cell level, a highly robust scRNA-seq pipeline was developed that highlighted potential chemokine mRNAs and microRNAs as biomarkers for early-stage lung cancer that might prompt the start of specific targeted therapies and possibly predict relapse (Kim et al., 2021). Additionally, another SCS study of patients with lung adenocarcinoma revealed a novel 10-gene prognostic risk model that achieved acceptable robustness and prediction accuracy in datasets from different platforms (Xu et al., 2022).

### 3.2 Integration into clinical molecular diagnostics

The integration of SCS into clinical molecular diagnostics is still in its early stages but is rapidly advancing due to its efficiency. Using SCS, healthcare providers can better understand cancer heterogeneity, leading to more accurate prognostic assessment, personalized treatment plans, and enhanced precision of cancer diagnosis. For example, pancreatic cancer is known for its poor prognosis, usually because it is clinically silent and diagnosed at a late stage. A 2023 SCS study detected overexpression of *GAS2L1* and *EPCAM* in circulating tumor cells and highlighted the potential of *GAS2L1* as a novel biomarker for the diagnosis of pancreatic cancer (Zhang et al., 2023). In terms of targeted therapy selection, information about the tumor heterogeneity obtained from SCS can be used to tailor the most appropriate treatment plan. For example, a novel computational predictive methodology called Beyondcell was successfully applied to various studies on cancer therapeutic heterogeneity involving cancer cell lines, primary cancer samples, and mouse-derived xenografts. Beyondcell successfully identified distinct cell subpopulations in both cell lines and patient samples and explored tumor heterogeneity to identify resistant and sensitive subpopulations. It was shown to predict immunotherapy responses in melanoma patients and, moreover, suggested drugs to overcome drug resistance in patients with lung cancer (Fustero-Torre et al., 2021). In terms of disease monitoring, a detailed, non-invasive disease monitoring approach can be developed to monitor disease progression or predict relapse. For example, in a study on babies with highly aggressive acute lymphoblastic leukemia, drug responses were analyzed by SCS in individual leukemic cells from bone marrow and blood samples. This enabled the differentiation of sensitive and resistant cells, thereby improving relapse prediction, together with the characteristics of therapy-resistant cells. The approach provided unique insights into relapse-initiating subpopulations by enhancing risk stratification at diagnosis (Candelli et al., 2022). Another study also demonstrated the benefits of SCS in improving risk stratification. Implementing SCS in individuals with multiple myeloma identified novel targets for therapy and enabled the development of a sophisticated stratification model called the 7-star model which, when combined with the standard ISS stage classification, effectively distinguished multiple myeloma patients at extremely high risk (Gong et al., 2022).

Beyond scRNA-seq, clinical single cell approaches now combine targeted scDNA panels with immunophenotyping to track measurable/minimal residual disease (MRD) (Demaree et al., 2021). After cancer treatments (e.g., chemotherapy), residual malignant cells can persist and lead to relapse, making their identification essential. Conventional assays (flow cytometry, bulk NGS) are insufficiently sensitive and are prone to generating misleading signals from other cell populations, especially since residual cells are often extremely rare and therefore masked by normal/benign cells. Single-cell methods allow the matching of genotype (DNA alterations) with phenotype (surface markers/immunophenotyping) in the same cell, increasing the certainty in identifying residual malignant cells (Robinson et al., 2023).

### 3.3 Future directions in oncology

SCS is poised to revolutionize cancer care. It promises to enhance precision medicine by enabling the development of highly personalized treatment plans that adapt to tumor evolution. It also promises to improve early detection and risk assessment, allowing for intervention before the cancer becomes clinically apparent or has spread. SCS is also expected to increase our understanding of tumor heterogeneity and in doing so reveal new therapeutic targets. It is expected to play a crucial role in identifying mechanisms of drug resistance, facilitating the creation of strategies to overcome or prevent them. SCS also has potential for discovering and validating new biomarkers for disease monitoring and treatment adjustment. Integrating SCS with other cutting-edge technologies like AI and spatial transcriptomics will offer a holistic view of cancer complexity (Nofech-Mozes et al., 2023). However, efforts are still underway to translate SCS technologies into clinical practice. For example, there is a need to streamline SCS data analysis and for more effective tools for oncology-related data analysis, which would make the clinical application of SCS more accessible to oncologists and facilitate their integration into routine cancer care.

## 4 Genetic and rare diseases

### 4.1 The role of SCS in rare diseases through detection of mosaicism and novel mutations

One important application of SCS is the detection of mosaicism, which refers to the presence of two or more genetically distinct cell populations derived from a single zygote. Germline mosaicism describes a mutation that specifically affects the gonads and can be inherited by offspring. Somatic mosaicism occurs when postzygotic mutation occurs early on, at any stage of soma development, or even in adult tissues (Thorpe et al., 2020). For example, most somatic mutations are either unique to 1 cell or present at very low mosaicism, which is why they cannot be detected by traditional DNA sequencing. However, scDNA-seq can recognize somatic mutations regardless of the degree of mosaicism (Evrony et al., 2021), helping to detect rare diseases caused by a mosaic mutation. For example, one study quantified a somatic brain mutation that causes mosaicism and hemimegalencephaly (Evrony et al., 2012). A sample was taken from a child with isolated hemimegalencephaly resulting from an E17K somatic missense point mutation in *AKT3*, present in the brain but not the blood. Using SCS, the authors detected the mutation not only in neuronal cells but also in non-neuronal cells, consistent with the patient’s MRI findings of both gray and white matter abnormalities (Poduri et al., 2012). These findings suggest that the mutation originated from an early neocortical progenitor that distributed throughout the hemisphere to give rise to both neuronal and non-neuronal cells.

Another example of the application of SCS is detecting rare cell populations. Genetic mutations or dysregulation may impact particular cells or subpopulations of cells in rare disorders. These rare cell populations can be detected in tissues or biological samples by SCS, providing the opportunity to examine their biological characteristics and understand how they contribute to disease pathology.

For example, one study profiled gene expression in nearly 10,000 individual liver cells from nine healthy donors (Aizarani et al., 2019). Previously unidentified subtypes of hepatocytes, Kupffer cells, and endothelial cells with distinct gene expression profiles were identified, together with a rare subset of bile duct cells with the ability to develop into organoids. This precursor cell type may be crucial for liver regeneration, since it could develop into bile duct cells or hepatocytes. This knowledge is important for understanding rare liver diseases that affect those sub-populations and advance current models for liver donation.

Finally, in rare disease diagnostics, when traditional exome or genome sequencing fails to identify many undiagnosed cases, SCS could also help to determine the cellular context of variants of unknown significance (VUS) and their associations with transcriptional or epigenetic states (Sreenivasan et al., 2022).

### 4.2 Precision diagnostics: integration of SCS into clinical molecular diagnostics for personalized insights into the genetic basis of rare diseases

SCS is showing promise for providing more information about rare diseases to guide their diagnosis and management. One study identified key genes in Moyamoya disease, a rare, long-term, occlusive cerebrovascular disorder with an unclear etiology defined by aberrant vascular networks at the base of the brain and steno-occlusive alterations in the internal carotid artery. This study identified heterogeneity in peripheral blood cells from patients with Moyamoya disease and implicated several genes in disease development, including *PTP4A1, SPINT2, CSTB, PLA2G16, GPX1, HN1, LGALS3BP, IFI6, NDRG1, GOLGA2*, and *LGALS3*. Furthermore, the approach clearly identified interactions between different cell types, providing a reference to understand the pathophysiology and crosstalk between cells and pathways. The BTLA-TNFRSF14 pathway was implicated in the disease, which may be a target for therapy. SCS not only helps to improve knowledge about the pathophysiology of a disease but also identifies target pathways for diagnosis and treatment (Tang et al., 2023).

### 4.3 Strategies for integrating SCS data with genetic counseling to inform patient management and family planning

The goal of genetic counseling is to inform and assist a person in understanding the psychological, physical, and familial complications of hereditary diseases. A broad range of techniques are included in the genetic counseling process, such as taking a family history and performing risk assessment, educating the public about a disorder and its inheritance patterns, reviewing available genetic testing options, and offering psychological support to help the patient adjust to the diagnosis or carrier status (Resta et al., 2006). A genetic counselor receives the genetic test results, interprets them, and then informs the patient or family about the results in a simple and understandable way. Informing patients properly with the results is important for decision-making in terms of life planning. The main goal of genetic counseling is to help individuals make informed choices by providing them with proper guidance.

SCS data offer knowledge about genetic variations that might affect gene expression in specific tissues, as many diseases have tissue-specific manifestations. Bulk sequencing techniques are limited to easily accessed tissues like skin and blood, which does not provide information about the genetic variability present in specific tissues that might be related to disease development. SCS, however, might overcome some of these limitations (Cuomo et al., 2023).

It is essential to combine genetic counseling with SCS data to advise patients and families with genetic disorders. This could be established using different strategies.

#### 4.3.1 Interpretation of SCS results

Genetic counselors must explain the results in an understandable way to help patients and their families. These results may have implications for subjects’ health and the likelihood of recurrence in future pregnancies.

#### 4.3.2 Personalized risk assessment

Genetic counselors could use SCS data and family history information to provide thorough personalized risk evaluations (Baptista, 2005). SCS data provide highly detailed genetic profiles - and therefore individual risk profiles - because different single cells are sequenced from the same tissue. Therefore, genetic counselors can assess the probability of recurrence for particular genetic diseases and offer tailored risk estimates to support patients in making family planning decisions.

#### 4.3.3 Discussion of reproductive options

One of the main goals for genetic counselors is to discuss reproductive options with patients while considering their unique needs and preferences. For example, they provide details on preimplantation genetic testing (PGT) for couples undergoing *in vitro* fertilization (IVF) and the available prenatal diagnostic procedures, including chorionic villus sampling (CVS) and amniocentesis (Lilienthal and Cahr, 2020). One study examined cortical development prenatally and postnatally using SCS, defining the cell types and developmental stages most enriched for genetic risk factors for neurodevelopmental diseases. In addition, they mapped a comprehensive transcriptomic atlas of cortical lineages across prenatal and postnatal development, highlighting sex-specific developmental changes. Their findings provided insights into the pathophysiology of autism, genetic susceptibilities to developmental brain diseases, and lineage-specific mechanisms of normal cortical development. This information might be useful for improving the diagnosis of neurodevelopmental diseases prenatally, such that the family can take appropriate decisions with respect to family planning (Velmeshev et al., 2023).

## 5 Infectious diseases

The benefits of SCS are not limited to studying cellular heterogeneity in humans, with extensive work also carried out to use the technology to study pathogen diversity (Yadav et al., 2023). The detailed genetic and molecular information provided by SCS is useful for providing a better understanding of the strains and types of microorganisms to enable broad approaches for investigating host-pathogen interactions that may impact drug utilization (Bawn et al., 2022). As genetic material alters over time, knowledge of the accumulation of mutations or other genetic alterations may answer several questions relating to prognosis and treatment selection. Pathogen heterogeneity may lead to a drug failure phenomenon known as hetero-resistance, where a subpopulation of pathogens is resistant to the medication and continues replicating (Band and Weiss, 2019). In addition, adaptation of pathogen subpopulations to certain host tissues or organs makes it challenging to detect them by conventional methods (Huang W. et al., 2021); indeed, traditional methods like bulk sequencing study pathogen populations and exclude heterogeneity within or between pathogen families. In contrast, SCS can fully characterize pathogen heterogeneity in patients based on the four pillars of pathogen differences, evolution tracking, understanding quorum sensing in pathogen-pathogen interactions, and identifying drug targets. One study examined host-pathogen interactions in distal lung epithelial cells, and scRNA-seq successfully detected subpopulation differences (Penaranda and Hung, 2019). These results suggest that SCS applications may change point-of-care procedures via tracking of microbial populations to generate more targeted therapies. SCS can be applied to prescribe drugs that target specific subpopulations guided by SCS data, selection of the most effective antimicrobial agents, optimizing drug doses, and monitoring treatment responses (Zhang et al., 2024).

### 5.1 Application of SCS to pathogen heterogeneity and host-pathogen interactions

Infection is a dynamic process that involves host-pathogen interactions. SCS enables the analysis of immune responses and cellular diversity in infectious diseases to understand the detail of the underlying mechanisms and inspire novel interventions. SCS has revolutionized the identification of rare cell populations within host-pathogen interactions (Penaranda and Hung, 2019) that might represent niches for pathogens through unique virulence factors or immune evasion mechanisms that impact disease severity or progression (See et al., 2018). SCS provides individual profiles and highly granular information on rare cell types like pathogen-infected host cells or specific immune cell subsets that might be orchestrating pathogen defense mechanisms. Also, discovering host-pathogen reactions using SCS in transient immune responses is significant for controlling infections, as current immunological tests may miss cells with short life spans (such as neutrophils) that are critical to infection responses (Heyman et al., 2023).

It has always been challenging to characterize the temporal dynamics of immune networks during infection. Traditional tools offer only snapshots of the infection, but SCS can capture all events simultaneously. Analyzing cells at different time points helps to reconstruct the event sequence and inform how cell types interact in terms of gene expression during infections. Sequencing-based technologies can help to clarify host interactions from recognition of the pathogen to the response, revealing unknown steps in early infection to develop preventative measures (Pan et al., 2022). In most cases, responses to medications are unclear, but clinicians can differentiate between cleared and chronic infections by knowing the clearance or persistence status using SCS, which allows for a detailed overview of signaling and immune networks. SCS is not restricted to assessing the state of infected cells, as combined sequencing tools enable the tracking of cell fate for both host and pathogen to list factors mediating pathogen resistance or immune exhaustion. One example used three-dimensional intact tissue sequencing of SC transcriptional states for immunological studies (Chen et al., 2019).

Heterogeneity profiles provided by SCS can be extended to include functional diversity. The immune system landscape involves production and differentiation of many cell types during infection, and cells are produced unequally with many specific functions. Single cell studies can determine cells with specific functions such as antigen presentation, cytokine secretion, and cytotoxic activity. SCS can also be used to elucidate the communication and coordination between immune cells to fight pathogens. High-dimensional SCS has identified organ-specific signatures and conserved natural killer (NK) cell subsets in humans and mice (Cao et al., 2020).

### 5.2 SCS in antimicrobial therapy: resistance and virulence factors

A new and growing application of SCS in immunology is for antimicrobial therapy, where it can be used to understand resistance mechanisms, study pathogens and their virulence factors, predict treatment response, identify novel drug targets, and monitor treatment efficacy. Microorganisms can defend themselves against host cells through the production of virulence factors, and these can also initiate resistance to antimicrobial drugs (Sharma et al., 2017). One emerging SC method is the scPAIR-seq approach, which is used to functionally analyze the effect of bacterial mutants on host immunity at SC resolution. This technique studies infection using a pooled library of multiplex-tagged, barcoded bacterial mutants by scRNA-seq. scPAIR-seq was applied to macrophages infected with a library of *Salmonella tryphimurim* secretion system effector mutants, and SCS revealed redundancy between the two populations with specific fingerprints. By mapping the global virulence network of individual effectors based on their impact on the host immune pathways, scPAIR-seq enables the study of the interplay between host and pathogen in any infection model and bacterial strain (Heyman et al., 2023).

### 5.3 Examples of vaccine development by SCS

Vaccine immunogenicity is monitored via many factors including the vaccine platform, antigen, and adjuvant. SCS characterizes these influences with high specificity and can be used to study heterogeneous responses in vaccine regimens. For instance, one study developed a personalized cancer vaccine using whole-genome sequencing combined with scRNA-seq from an acute myeloid leukemia patient, which differentiated wild type from tumor cells. It also detected abnormal tumor cells and pinpointed mutations in the transcriptional profile.

Another study sought to generate a hepatitis B vaccine using SCS. Around 5%–10% of vaccinated people lack immunity to the virus, at least in part due to heterogeneity at the cellular level regulating responses. Typically, population signals masking cellular signals result in low vaccine efficacy. scRNA-seq was used to sequence peripheral blood mononuclear cells (PBMCs) from high immunity and no immunity individuals. Antigen activity and cytotoxicity scores were measured for the two groups, which revealed that mitogen-activated protein kinase (MAPK) activity scores for naïve B cells and effector T cells were dramatically decreased, while cytotoxicity scores for NK cells significantly increased, in the no immunity compared with the high immunity group (Zhao et al., 2023).

### 5.4 Opportunities and challenges for SCS in the diagnosis of infectious diseases

A major challenge to integrating SCS into clinical research is data complexity. Extracting meaningful insights from complex datasets requires advanced bioinformatic tools and expertise. While data collection might be feasible, its interpretation is another issue. The ability to distinguish true rare populations with sufficient evidence needs robust tools and experimental validation (Penaranda and Hung, 2019). Protocols should select the most informative time for sample collection, preparation, and analysis to fully represent the event. Robust, QC-assured SCS methods must be established to be accepted clinically and to generate reliable data (Lin et al., 2020).

SCS is therefore showing promise for the diagnosis and management of infectious diseases. Targeted therapies for subpopulations with essential functions in pathogen resistance could be administered based on SCS-derived biomarkers to improve precision medicine (Wiedmeier et al., 2019). SCS allows for the highly specific identification of individual pathogens within a complex mix of different strains and selecting the most effective treatment, leading to a more targeted antibiotic choice and potentially reducing the emergence of resistant strains. By integrating SCS into diagnostic tools, healthcare professionals can track how pathogens evolve and spread in real-time for early detection of outbreaks and to implement effective containment measures. SCS data offer insights into a patient’s immune response, how the pathogen resists treatment, and its overall virulence (Vallejos et al., 2017). This information is critical for selecting optimal antibiotics and even developing next-generation anti-virulence therapies specifically tailored to the unique characteristics of the individual’s infection.

## 6 Autoimmune disorders and inflammation

Autoimmune inflammatory diseases represent a group of disorders of the immune system that exhibit considerable clinical and pathophysiological heterogeneity (Kuret et al., 2022). This heterogeneity makes translational studies on immune-mediated diseases challenging. Genetic variability is a significant contributor to this heterogeneity. For example, human leukocyte antigen (HLA) genes, among other inherited genetic risk variants, create variability in immune system responses. Similarly, environmental triggers are another source of variability, and infections or chemical exposures represent external factors that can exacerbate autoimmune reactions. Furthermore, a breakdown in immune tolerance also contributes to autoimmune pathogenesis (Sulen et al., 2020). Traditional bulk techniques are also limited by the number of immune cells in some samples, small tissue samples, and the presence of rare cell populations, which require a large number of cells defined by a limited set of markers (Sharma et al., 2018).

SCS is now considered to be a robust tool that has revealed some of the complex immunoregulatory pathways and immune responses underlying autoimmune disorders and inflammation (Sharma et al., 2018; He et al., 2022). Furthermore, researchers have used SCS to identify druggable targets and pathways together with novel biomarkers. This improved knowledge can lead to enhanced classification of disease, early detection of illness, assessment of the treatment responses, and personalized therapeutic strategies (Sharma et al., 2018).

### 6.1 Identification of biomarkers for disease activity and treatment response

As discussed above, scRNA-seq is useful for studying systems characterized by high cellular heterogeneity, such as the immune system (Kuret et al., 2022). In systemic sclerosis (SSc), for example, specific gene expression profiles in lung fibroblasts and myofibroblasts provide insights into the mechanisms of development of interstitial lung disease in SSc. For example, myofibroblasts expressing high levels of *ACTA2* have been linked to fibrosis, while subsets of macrophages (expressing *SP1*, *FABP4*, and *FCN1*) have been associated with immune responses in SSc. Additionally, the presence of specific cell populations in skin tissues, such as myeloid cell populations with high expression of chemokines like *CXCL13*, suggests specific underlying inflammatory mechanisms in SSc-related skin fibrosis. In psoriatic arthritis, scRNA-seq has identified a unique profile of monocyte/macrophages in synovial fluid and a potential therapeutic target through the tryptase-6PAR2 signaling pathway. Similarly, in axial spondylarthrosis (axSpA), scRNA-seq revealed expansion of mature GZMB^+^ T cells and a distinct interferon (IFN) signature that distinguishes this condition from Crohn’s disease (CD) and combined CD-axSpA cases (Kuret et al., 2022). These unique patterns discovered by scRNA-seq can help guide personalized treatment decisions.

### 6.2 Illustrating the application of SCS in clinical research and diagnostics for autoimmune conditions

SCS has provided valuable insights into several autoimmune disorders. The following discussion illustrates the diverse applications of SCS to clinical research and diagnostics for autoimmune neurological disorders and rheumatic diseases.

#### 6.2.1 Neuromyelitis optica spectrum disorder (NMOSD)

NMOSD involves autoantibodies targeting aquaporine-4 (AQP4), which lead to central nervous system (CNS) damage. SCS has been used to determine whether anti-AQP4 antibodies originate from peripheral B cells or CNS-resident B cells. After constructing transcriptomic libraries from cerebrospinal fluid (CSF), researchers found a significant proportion of anti-AQP4 sequences in CSF, suggesting intrathecal production by CNS-resident plasma cells (Zou A. et al., 2021). This discovery enhances understanding of the pathogenesis of NMOSD that could potentially guide targeted immunotherapeutic strategies.

#### 6.2.2 Anti-NMDA receptor encephalitis (NMDA)

Anti-N-methyl-D-aspartate receptor encephalitis is another autoimmune neurological disorder where antibodies target NMDA receptors in the brain, causing severe neurological symptoms. One of the key challenges in studying this disorder is the polyclonal feature of serum and CSF. SCS has overcome this challenge by analyzing the transcriptomes of individual B cells and characterizing their subsets (through clonality and somatic hypermutation patterns) involved in autoantibody production (Zou A. et al., 2021). This has provided insights into the origin of these B cell subtypes and linked them to disease activity.

#### 6.2.3 Myasthenia gravis (MG)

MG is caused by autoantibodies that target the neuromuscular junction, resulting in muscle weakness. SCS has allowed researchers to identify the B cell subsets responsible for autoantibody production and analyze clonal diversity and affinity maturation. By knowing the specific B cell population involved in disease pathophysiology, precise immunotherapies can be developed (Zou A. et al., 2021).

#### 6.2.4 Multiple sclerosis (MS)

MS is characterized by inflammatory demyelinating lesions in the CNS affecting the spinal cord, brain, and optic nerve, and it is mainly associated with damage to white matter. Oligodendrocytes, the cells responsible for myelin sheath formation, are central to MS pathogenesis. SCS has allowed the characterization of oligodendrocyte heterogeneity. SCS detected 12 oligodendrocytes subtypes, from precursors to mature cells, in mice and discovered a new cell population in blood vessels distinct from traditional oligodendrocytes. The new understanding that some oligodendrocytes differentiate to form myelin while others create fine networks could lead to innovative myelin regeneration strategies.

Astrocytes also contribute to MS pathogenesis. SCS has shown that mouse and human astrocytes are transcriptionally heterogeneous, and some can enhance inflammation in the CNS through the *MAFG* gene, which may act as a biomarker for targeted therapies for MS patients. In addition, microglia maintain CNS homeostasis and their activation under injury. SCS revealed that some subsets of microglia were more active than others in contributing to disease processes. These findings open up new avenues for more targeted treatments in the future (Zhao et al., 2021).

#### 6.2.5 Rheumatoid arthritis (RA)

RA is a chronic autoimmune disease characterized by persistent inflammation and progressive joint damage. The application of scRNA-seq to RA has enhanced understanding of the cellular and molecular landscape of this disease. By examining individual cells within inflamed synovial tissue and adjacent lymph nodes, scRNA-seq revealed the complexity and heterogeneity of RA. Moreover, scRNA-seq has identified specific gene expression patterns associated with disease activity and remission, with distinct macrophage subpopulations and fibroblast subtypes serving as potential biomarkers of inflammation and tissue damage (Kuret et al., 2022; Zhao et al., 2021). In a mouse model of antigen-induced arthritis (AIA), scRNA-seq identified pathways related to T cell differentiation, corroborating current understanding of RA pathogenesis and stressing the importance of targeting pathways using existing drugs. The construction of a multicellular disease model using scRNA-seq data has also demonstrated complex interactions between various cell types in RA, indicating that multiple mechanisms contribute to disease progression. While this complicates drug discovery, the interconnectivity between cell types could offer diagnostic advantages. For instance, analyses of T cells from the peripheral blood of RA patients show potential as diagnostic features. Additionally, T cell expression profiles can accurately differentiate RA from other immune-related diseases. This offers a promising approach using SCS for early diagnosis and personalized treatment strategies (Gawel et al., 2019). In addition, unique characteristics of T cell receptor beta in CD8^+^ T cells have been identified associated with disease aggression. Hence, SCS holds the potential to develop improved protocols for resistance therapy in patients with RA (He et al., 2022).

#### 6.2.6 Systemic lupus erythematosus (SLE)

scRNA-seq in systemic lupus erythematosus (SLE) has revealed an interferon (IFN) response signature in various tissues, including kidney and skin, which correlates with disease severity and responses to therapy. The detected IFN signature, along with the expression of chemokine receptors like CXCR4 and CX3CR1, was a potential biomarker for lupus nephritis and other SLE complications. Additionally, scRNA-seq has revealed specific immune cell populations and signaling pathways that could potentially serve as indicators of treatment response. For example, the presence of specific B cell subpopulations and regulatory eosinophils in RA and SLE has been linked to therapeutic outcomes (Kuret et al., 2022; Zou A. et al., 2021). In addition, analysis of 16 immune cell types in the peripheral blood revealed an increase in T and B cell receptor types and an immune response signature in SLE cases that can be utilized to accurately diagnose and precisely treat SLE patients (He et al., 2022). SCS analysis of examined kidney, urine, and blood samples from lupus nephritis patients revealed that urine-derived cells could serve as an alternative and less invasive sample to kidney biopsies with respect to analyzing the molecular activation states of specific leukocytes subsets, potentially offering new ways to monitor disease activity and progression (Zou A. et al., 2021; Wajda et al., 2021).

#### 6.2.7 Other conditions

Single-cell T cell receptor sequencing revealed specific T cell receptor alpha and beta motifs in Th1 and Th17 cells in primary Sjogren’s syndrome. In adenosine deaminase two deficiency, SCS identified activation of CD8^+^ and CD4^+^ T cells and cell-cell interactions between T cells and monocytes, leading to upregulation of STAT1 expression in T cells. In autoimmune hepatitis, SCS identified specific autoreactive CD4^+^ T cells and a potential correlation between the immune response and soluble liver antigen reactivity. SCS has shown that Graves’ hyperthyroidism is associated with clonal expansion of CD4^+^ cytotoxic T lymphocytes, potentially in recurrent disease (He et al., 2022). Moreover, SCS has been used to study islet cells and immune cell interactions in type 1 diabetes to try to identify the factors contributing to the autoimmune response against pancreatic beta cells and new biomarkers for early detection of the disease. IL-32 was found to be upregulated by activated T cells and NK cells before the appearance of autoantibodies typically associated with type 1 diabetes. This early increase in IL-32 might be an early biomarker for detecting abnormal immune function before type 1 diabetes becomes clinically apparent. Another biomarker identified using SCS was expanded T cell receptor clonotypes in islet antigen-reactive CD4^+^ memory T cells, which could serve as biomarkers for disease progression and targets for antigen-specific therapies (Zou A. et al., 2021).

### 6.3 The future of SCS in personalized treatment plans for autoimmune disorders

Recent single-cell studies have revealed previously unrecognized immune heterogeneity, highlighting future transformative applications in precision immunology. For example, scRNA-seq profiling of acute respiratory distress syndrome identified alveolar IL-1β^hi^ neutrophils as key amplifiers of inflammation through macrophage-neutrophil feedback loops. This suggests that similar cellular circuits could be mapped to understand tissue-specific inflammatory progression (Yang et al., 2025). Likewise, single-cell and multiplex imaging analyses in Ig4-related disease uncovered novel immune subsets such as GZMK + cytotoxic Tfh cells, MKI67 + B cells, and double-negative type 3 B cells, together with their interactions, involved in the immunopathogenesis of Ig4-related disease across different clinical phenotypes (Hara, 2025). Similarly, single-cell transcriptomic profiling of double-negative (DNT) T cells demonstrated that some populations are pro-inflammatory, while others are regulatory or suppressive, thereby deepening our understanding of immune dysregulation that might guide the development of targeted, cell-specific strategies (Li et al., 2025).

These insights underscore the importance of a personalized approach to therapy in autoimmune diseases. The traditional “one-size-fits-all” model is widely regarded as ineffective, leading to drug failure and reduced quality of life (Kuret et al., 2022). By identifying specific biomarkers for disease activity and treatment responses, single-cell technologies could guide more informed and patient-centered treatment strategies. Future advances in SCS technologies are expected to further enhance the precision of autoimmune disorder diagnosis and treatment, thereby optimizing patient care.

## 7 Challenges and future directions in SCS

### 7.1 Current challenges

SCS is an excellent method for visualizing gene expression in various types of cells at high resolution. However, some challenges might limit its use.

SCS is considered a more complex process than bulk sequencing. It requires cell sorting to separate populations of cells required for the study, extending the sample processing time. In addition, SCS relies on detecting the small amount of DNA/RNA molecules in each cell, resulting in low coverage (Ascensión et al., 2022). While amplification is useful, it can increase the background noise, introduce errors, and increase amplification bias (Sreenivasan et al., 2022).

Moreover, SCS suffers from high variability, and gene expression levels can significantly differ between similar cell types. It is worth mentioning that time and conservation media can also affect gene expression and introduce bias; for example, gene expression is lower samples processed late after extraction (Massoni-Badosa et al., 2020).

SCS produces complex data that must be accurately analyzed by an expert in biology and data analysis. Data from SCS must undergo a series of analytic steps such data normalization and dimensionality reduction. Several techniques have been developed to normalize data from SCS, since bulk-based normalization techniques have been found to be inappropriate due to the high level of technical noise (Yip et al., 2017).

Another problem associated with scRNA-seq data is high dimensionality. Therefore, it is important to reduce the collection of random variables and utilize the primary variables that provide a comprehensive description of the data (Adil et al., 2021).

Finally, many analyses still need the discretion and interpretation of a skilled bioinformatician as they are not fully standardized (Lähnemann et al., 2020). In addition to the technical limitations of SCS, other obstacles stand in the way of its clinical implementation. For example, there are legitimate ethical and privacy concerns related to the sensitive genomic information arising from SCS (Walker et al., 2024). Also, uneven access to modern sequencing infrastructure, particularly in low- and middle-income countries, exacerbates worldwide inequities in data generation and research capabilities (Boakye et al., 2024). The diverse knowledge required to analyze and interpret SCS data underscores the need for increased training and workforce development in computational and translational genomics (Overbey et al., 2022). Finally, cost-effectiveness remains a major obstacle, as the infrastructure and computational resources required for SCS make it inaccessible, particularly in low- and middle-income nations. Furthermore, gaps in worldwide research capability lead to uneven data representation, while challenges of data storage, sharing, and the ethical use of sensitive genomic information continue to hinder the routine clinical deployment of SCS.

### 7.2 Future directions

It is anticipated that the cost per SCS sample will continue to decrease, coupled with increasing cellular throughput and the ability to multiplex samples during the barcoding process. To address the issues raised by limited data from dropout and data variability between batches, computational techniques should evolve and become fully automated and integrated into instruments. Automated instruments will help to standardize SCS procedures currently performed by domain experts.

Reducing or even eliminating DNA or RNA amplification before sequencing has the potential to improve the accuracy of single-cell analysis and make it more reliable.

Moreover, it is important to develop powerful platforms and software for SCS data analysis, as data interpretation remains a huge challenge in practical SCS.

## 8 Conclusion

SCS technologies have transformed clinical molecular diagnostics by increasing understanding of cell-to-cell variability, disease pathogenesis, and therapy responses. This unique view of cells has been useful in the accurate, sensitive, and specific analysis of several diseases, including but not limited to cancer, autoimmunity, rare genetic conditions, and infectious diseases. The possibilities outlined by the single-cell approach will enable treatment strategies tailored to the distinct cellular profile of each patient.

Despite the significant potential of SCS, integrating it into clinical practice comes with various challenges, such as complexity of data analysis, a lack of standardization, and high costs. Collaboration between clinicians, molecular biologists, bioinformaticians, and other healthcare professionals is essential to overcome these hurdles. Efforts to establish and diversify standards and develop rigorous protocols are critical to enhance the accessibility and reliability of SCS into daily clinical use. Given the data complexity, the application of sophisticated bioinformatics tools and advanced analysis will require cooperation and innovative commitment.

The potential of SCS for clinical molecular diagnostics is promising, with prospects for the identification of new biomarkers, predicting treatment responses, monitoring disease progression, and helping with therapy decision-making. As technology continues to advance, precision and personalization in medicine are expected to increase. The development of improved data analysis tools, automated instruments, and comprehensive validation studies will further improve the seamless integration of SCS into daily clinical practice.

In conclusion, the successful implementation of SCS in real-world clinical scenarios would necessitate continued investigation, cooperation across stakeholders, as well as evidence-based efforts to overcome technological, logistical, and resourcing challenges. Patient-centered care coupled with SCS promises to fundamentally change the process by which we diagnose, treat, and control various disorders, thereby enhancing patient outcomes.

## References

[B1] Adhit K. K. Wanjari A. Menon S. K S. (2023). Liquid biopsy: an evolving paradigm for non-invasive disease diagnosis and monitoring in medicine. Cureus 15, e50176. 10.7759/cureus.50176 38192931 PMC10772356

[B2] Adil A. Kumar V. Jan A. T. Asger M. (2021). Single-cell transcriptomics: current methods and challenges in data acquisition and analysis. Front. Neurosci. 15, 591122. 10.3389/fnins.2021.591122 33967674 PMC8100238

[B3] Ahmed R. Zaman T. Chowdhury F. Mraiche F. Tariq M. Ahmad I. S. (2022). Single-Cell RNA sequencing with spatial transcriptomics of cancer tissues. IJMS 23, 3042. 10.3390/ijms23063042 35328458 PMC8955933

[B4] Aizarani N. Saviano A. Sagar null Mailly L. Durand S. Herman J. S. (2019). A human liver cell atlas reveals heterogeneity and epithelial progenitors. Nature 572, 199–204. 10.1038/s41586-019-1373-2 31292543 PMC6687507

[B5] Alessandrì L. Arigoni M. Calogero R. (2019). “Differential expression analysis in single-cell transcriptomics,” in *Single cell methods*. Methods in molecular biology. Editor Proserpio V. (New York, NY: Springer New York), 425–432. 10.1007/978-1-4939-9240-9_25 31028652

[B6] Ascensión A. M. Araúzo-Bravo M. J. Izeta A. (2022). Challenges and opportunities for the translation of single-cell RNA sequencing technologies to dermatology. Life (Basel) 12, 67. 10.3390/life12010067 35054460 PMC8781146

[B7] Band V. I. Weiss D. S. (2019). Heteroresistance: a cause of unexplained antibiotic treatment failure? PLoS Pathog. 15, e1007726. 10.1371/journal.ppat.1007726 31170271 PMC6553791

[B8] Baptista P. V. (2005). Principles in genetic risk assessment. Ther. Clin. Risk Manag. 1, 15–20. 10.2147/tcrm.1.1.15.53606 18360538 PMC1661604

[B9] Bawn M. Hernandez J. Trampari E. Thilliez G. Quince C. Webber M. A. (2022). Single-cell genomics reveals population structures from *in vitro* evolutionary studies of salmonella. Microb. Genomics 8, mgen000871. 10.1099/mgen.0.000871 36125951 PMC9676037

[B10] Bischoff P. Trinks A. Obermayer B. Pett J. P. Wiederspahn J. Uhlitz F. (2021). Single-cell RNA sequencing reveals distinct tumor microenvironmental patterns in lung adenocarcinoma. Oncogene 40, 6748–6758. 10.1038/s41388-021-02054-3 34663877 PMC8677623

[B11] Boakye S. T. Cribbs A. P. Baldwin M. J. Masimirembwa C. Chikwambi Z. Kerasidou A. (2024). Overcoming barriers to single-cell RNA sequencing adoption in low- and middle-income countries. Eur. J. Hum. Genet. 32, 1206–1213. 10.1038/s41431-024-01564-4 38565638 PMC11499908

[B12] Briones J. Espulgar W. Koyama S. Takamatsu H. Tamiya E. Saito M. (2021). A design and optimization of a high throughput valve based microfluidic device for single cell compartmentalization and analysis. Sci. Rep. 11, 12995. 10.1038/s41598-021-92472-w 34155296 PMC8217553

[B13] Candelli T. Schneider P. Garrido Castro P. Jones L. A. Bodewes E. Rockx-Brouwer D. (2022). Identification and characterization of relapse-initiating cells in MLL-Rearranged infant ALL by single-cell transcriptomics. Leukemia 36, 58–67. 10.1038/s41375-021-01341-y 34304246 PMC8727302

[B14] Cao Y. Qiu Y. Tu G. Yang C. (2020). Single-cell RNA sequencing in immunology. CG 21, 564–575. 10.2174/1389202921999201020203249 33414678 PMC7770633

[B15] Chen H. Ye F. Guo G. (2019). Revolutionizing immunology with single-cell RNA sequencing. Cell Mol. Immunol. 16, 242–249. 10.1038/s41423-019-0214-4 30796351 PMC6460502

[B16] Choi Y. H. Kim J. K. (2019). Dissecting cellular heterogeneity using single-cell RNA sequencing. Mol. Cells 42, 189–199. 10.14348/molcells.2019.2446 30764602 PMC6449718

[B17] Chu X. Tian W. Ning J. Xiao G. Zhou Y. Wang Z. (2024). Cancer stem cells: advances in knowledge and implications for cancer therapy. Signal Transduct. Target Ther. 9, 170. 10.1038/s41392-024-01851-y 38965243 PMC11224386

[B18] Clark I. C. Delley C. L. Sun C. Thakur R. Stott S. L. Thaploo S. (2020). Targeted single-cell RNA and DNA sequencing with fluorescence-activated droplet merger. Anal. Chem. 92, 14616–14623. 10.1021/acs.analchem.0c03059 33049138 PMC8182774

[B19] Cuomo A. S. E. Nathan A. Raychaudhuri S. MacArthur D. G. Powell J. E. (2023). Single-cell genomics meets human genetics. Nat. Rev. Genet. 24, 535–549. 10.1038/s41576-023-00599-5 37085594 PMC10784789

[B20] Danilenko M. Clifford S. C. Schwalbe E. C. (2021). Inter and intra-tumoral heterogeneity as a platform for personalized therapies in medulloblastoma. Pharmacol. Ther. 228, 107828. 10.1016/j.pharmthera.2021.107828 33662447

[B21] Demaree B. Delley C. L. Vasudevan H. N. Peretz C. A. C. Ruff D. Smith C. C. (2021). Joint profiling of DNA and proteins in single cells to dissect genotype-phenotype associations in leukemia. Nat. Commun. 12, 1583. 10.1038/s41467-021-21810-3 33707421 PMC7952600

[B22] Deng Y. Guo Y. Xu B. (2020). Recent development of microfluidic technology for cell trapping in single cell analysis: a review. Processes 8, 1253. 10.3390/pr8101253

[B23] Egeblad M. Nakasone E. S. Werb Z. (2010). Tumors as organs: complex tissues that interface with the entire organism. Dev. Cell 18, 884–901. 10.1016/j.devcel.2010.05.012 20627072 PMC2905377

[B24] Evrony G. D. Cai X. Lee E. Hills L. B. Elhosary P. C. Lehmann H. S. (2012). Single-Neuron sequencing analysis of L1 retrotransposition and somatic mutation in the human brain. Cell 151, 483–496. 10.1016/j.cell.2012.09.035 23101622 PMC3567441

[B25] Evrony G. D. Hinch A. G. Luo C. (2021). Applications of single-cell DNA sequencing. Annu. Rev. Genomics Hum. Genet. 22, 171–197. 10.1146/annurev-genom-111320-090436 33722077 PMC8410678

[B26] Frąszczak K. Barczyński B. (2024). Characteristics of cancer stem cells and their potential role in endometrial cancer. Cancers (Basel) 16, 1083. 10.3390/cancers16061083 38539419 PMC10969160

[B27] Fustero-Torre C. Jiménez-Santos M. J. García-Martín S. Carretero-Puche C. García-Jimeno L. Ivanchuk V. (2021). Beyondcell: targeting cancer therapeutic heterogeneity in single-cell RNA-seq data. Genome Med. 13, 187. 10.1186/s13073-021-01001-x 34911571 PMC8675493

[B28] Gawel D. R. Serra-Musach J. Lilja S. Aagesen J. Arenas A. Asking B. (2019). A validated single-cell-based strategy to identify diagnostic and therapeutic targets in complex diseases. Genome Med. 11, 47. 10.1186/s13073-019-0657-3 31358043 PMC6664760

[B29] Gong L. Lv J. Qiu L. Sun H. Hao M. (2022). Identifying the cells of origin and risk-stratification strategy by single-cell sequencing in multiple myeloma. Blood 140, 12473. 10.1182/blood-2022-159485

[B30] Gross A. Schoendube J. Zimmermann S. Steeb M. Zengerle R. Koltay P. (2015). Technologies for single-cell isolation. IJMS 16, 16897–16919. 10.3390/ijms160816897 26213926 PMC4581176

[B31] Gu M. He T. Yuan Y. Duan S. Li X. Shen C. (2021). Single-Cell RNA sequencing reveals multiple pathways and the tumor microenvironment could lead to chemotherapy resistance in cervical cancer. Front. Oncol. 11, 753386. 10.3389/fonc.2021.753386 34900703 PMC8662819

[B32] Han Y. Wang D. Peng L. Huang T. He X. Wang J. (2022). Single-cell sequencing: a promising approach for uncovering the mechanisms of tumor metastasis. J. Hematol. Oncol. 15, 59. 10.1186/s13045-022-01280-w 35549970 PMC9096771

[B33] Haque A. Engel J. Teichmann S. A. Lönnberg T. (2017). A practical guide to single-cell RNA-sequencing for biomedical research and clinical applications. Genome Med. 9, 75. 10.1186/s13073-017-0467-4 28821273 PMC5561556

[B34] Hara S. (2025). Immunopathogenesis of IgG4-related disease in the era of single-cell RNA sequencing and highly multiplex immunofluorescence. Expert Rev. Clin. Immunol. 21, 1383–1401. 10.1080/1744666X.2025.2567589 40999941

[B35] Hayrabedyan S. Kostova P. Zlatkov V. Todorova K. (2021). Single-cell transcriptomics in the context of long-read nanopore sequencing. Biotechnol. and Biotechnol. Equip. 35, 1439–1451. 10.1080/13102818.2021.1988868

[B36] He J. Shen J. Luo W. Han Z. Xie F. Pang T. (2022). Research progress on application of single-cell TCR/BCR sequencing technology to the tumor immune microenvironment, autoimmune diseases, and infectious diseases. Front. Immunol. 13, 969808. 10.3389/fimmu.2022.969808 36059506 PMC9434330

[B37] Heyman O. Yehezkel D. Ciolli Mattioli C. Blumberger N. Rosenberg G. Solomon A. (2023). Paired single-cell host profiling with multiplex-tagged bacterial mutants reveals intracellular virulence-immune networks. Proc. Natl. Acad. Sci. U. S. A. 120, e2218812120. 10.1073/pnas.2218812120 37399397 PMC10334762

[B38] Hong R. Koga Y. Bandyadka S. Leshchyk A. Wang Y. Akavoor V. (2022). Comprehensive generation, visualization, and reporting of quality control metrics for single-cell RNA sequencing data. Nat. Commun. 13, 1688. 10.1038/s41467-022-29212-9 35354805 PMC8967915

[B39] Hu P. Zhang W. Xin H. Deng G. (2016). Single cell isolation and analysis. Front. Cell Dev. Biol. 4, 116. 10.3389/fcell.2016.00116 27826548 PMC5078503

[B40] Hu Y. Shen F. Yang X. Han T. Long Z. Wen J. (2023). Single-cell sequencing technology applied to epigenetics for the study of tumor heterogeneity. Clin. Epigenet. 15, 161. 10.1186/s13148-023-01574-x 37821906 PMC10568863

[B41] Huang W. Wang D. Yao Y.-F. (2021a). Understanding the pathogenesis of infectious diseases by single-cell RNA sequencing. Microb. Cell 8, 208–222. 10.15698/mic2021.09.759 34527720 PMC8404151

[B42] Huang R. H. Wang L. X. He J. Gao W. (2021b). Application and prospects of single cell sequencing in tumors. Biomark. Res. 9, 88. 10.1186/s40364-021-00336-2 34895349 PMC8665603

[B43] Illumina (2014). Illumina | sequencing and array solutions to fuel genomic discoveries. Available online at: https://www.illumina.com/ (Accessed May 2, 2024).

[B44] Jia Q. Chu H. Jin Z. Long H. Zhu B. (2022). High-throughput single-сell sequencing in cancer research. Sig. Transduct. Target Ther. 7, 145. 10.1038/s41392-022-00990-4 PMC9065032 35504878

[B45] Kidess E. Jeffrey S. S. (2013). Circulating tumor cells *versus* tumor-derived cell-free DNA: rivals or partners in cancer care in the era of single-cell analysis? Genome Med. 5, 70. 10.1186/gm474 23953663 PMC3979136

[B46] Kim C. Gao R. Sei E. Brandt R. Hartman J. Hatschek T. (2018). Chemoresistance evolution in triple-negative breast cancer delineated by single-cell sequencing. Cell 173, 879–893. 10.1016/j.cell.2018.03.041 29681456 PMC6132060

[B47] Kim J. Xu Z. Marignani P. A. (2021). Single-cell RNA sequencing for the identification of early-stage lung cancer biomarkers from circulating blood. npj Genom. Med. 6, 87. 10.1038/s41525-021-00248-y 34654834 PMC8519939

[B48] Korkusuz P. Köse S. Yersal N. Önen S. (2019). Magnetic-Based cell isolation technique for the selection of stem cells. Methods Mol. Biol. 1879, 153–163. 10.1007/7651_2018_151 30306535

[B49] Kuipers J. Jahn K. Beerenwinkel N. (2017). Advances in understanding tumour evolution through single-cell sequencing. Biochimica Biophysica Acta (BBA) - Rev. Cancer 1867, 127–138. 10.1016/j.bbcan.2017.02.001 28193548 PMC5813714

[B50] Kuksin M. Morel D. Aglave M. Danlos F.-X. Marabelle A. Zinovyev A. (2021). Applications of single-cell and bulk RNA sequencing in onco-immunology. Eur. J. Cancer 149, 193–210. 10.1016/j.ejca.2021.03.005 33866228

[B51] Kuret T. Sodin-Šemrl S. Leskošek B. Ferk P. (2022). Single cell RNA sequencing in autoimmune inflammatory rheumatic diseases: current applications, challenges and a step toward precision medicine. Front. Med. 8, 822804. 10.3389/fmed.2021.822804 35118101 PMC8804286

[B52] Lähnemann D. Köster J. Szczurek E. McCarthy D. J. Hicks S. C. Robinson M. D. (2020). Eleven grand challenges in single-cell data science. Genome Biol. 21, 31. 10.1186/s13059-020-1926-6 32033589 PMC7007675

[B53] Lakkis J. Wang D. Zhang Y. Hu G. Wang K. Pan H. (2021). A joint deep learning model enables simultaneous batch effect correction, denoising, and clustering in single-cell transcriptomics. Genome Res. 31, 1753–1766. 10.1101/gr.271874.120 34035047 PMC8494213

[B54] Li X. Wang C.-Y. (2021). From bulk, single-cell to spatial RNA sequencing. Int. J. Oral Sci. 13, 36. 10.1038/s41368-021-00146-0 34782601 PMC8593179

[B55] Li Y. Ma L. Wu D. Chen G. (2021). Advances in bulk and single-cell multi-omics approaches for systems biology and precision medicine. Briefings Bioinforma. 22, bbab024. 10.1093/bib/bbab024 33778867

[B56] Li Y. Wu S. Bai F. (2018). Molecular characterization of circulating tumor cells—from bench to bedside. Seminars Cell and Dev. Biol. 75, 88–97. 10.1016/j.semcdb.2017.09.013 28899718

[B57] Li X. Guo D. Zou I. X. Zhao L. Yang N. Liu Y. (2025). CD3+CD4-CD8- T cells: a new potential therapeutic target in treating autoimmune diseases. Front. Immunol. 16, 1683418. 10.3389/fimmu.2025.1683418 41080560 PMC12510931

[B58] Lilienthal D. Cahr M. (2020). Genetic counseling and assisted reproductive technologies. Cold Spring Harb. Perspect. Med. 10, a036566. 10.1101/cshperspect.a036566 31570374 PMC7605230

[B59] Lin W. N. Tay M. Z. Lu R. Liu Y. Chen C.-H. Cheow L. F. (2020). The role of single-cell technology in the Study and control of infectious diseases. Cells 9, 1440. 10.3390/cells9061440 32531928 PMC7348906

[B60] Liu B. Hu X. Feng K. Gao R. Xue Z. Zhang S. (2021). Temporal single-cell tracing reveals clonal revival and expansion of precursor exhausted T cells during anti-PD-1 therapy in lung cancer. Nat. Cancer 3, 108–121. 10.1038/s43018-021-00292-8 35121991

[B61] Maleki E. H. Bahrami A. R. Matin M. M. (2024). Cancer cell cycle heterogeneity as a critical determinant of therapeutic resistance. Genes and Dis. 11, 189–204. 10.1016/j.gendis.2022.11.025 37588236 PMC10425754

[B62] Mannarapu M. Dariya B. Bandapalli O. R. (2021). Application of single-cell sequencing technologies in pancreatic cancer. Mol. Cell Biochem. 476, 2429–2437. 10.1007/s11010-021-04095-4 33599893 PMC8119256

[B63] Massoni-Badosa R. Iacono G. Moutinho C. Kulis M. Palau N. Marchese D. (2020). Sampling time-dependent artifacts in single-cell genomics studies. Genome Biol. 21, 112. 10.1186/s13059-020-02032-0 32393363 PMC7212672

[B64] Mazutis L. Gilbert J. Ung W. L. Weitz D. A. Griffiths A. D. Heyman J. A. (2013). Single-cell analysis and sorting using droplet-based microfluidics. Nat. Protoc. 8, 870–891. 10.1038/nprot.2013.046 23558786 PMC4128248

[B65] Mi L.-Y. Gao J.-F. Ma D. Zhang L.-Y. Zhang G.-L. Xu K. (2021). Application of single-cell sequencing in autoimmune diseases. Chin. Med. J. 134, 495–497. 10.1097/CM9.0000000000001050 32889911 PMC7909166

[B66] Montgomery S. B. Bernstein J. A. Wheeler M. T. (2022). Toward transcriptomics as a primary tool for rare disease investigation. Cold Spring Harb. Mol. Case Stud. 8, a006198. 10.1101/mcs.a006198 35217565 PMC8958920

[B67] Mossner M. Baker A.-M. C. Graham T. A. (2021). The role of single-cell sequencing in studying tumour evolution. Fac. Rev. 10, 49. 10.12703/r/10-49 34131659 PMC8170688

[B68] Nath A. Bild A. H. (2021). Leveraging single-cell approaches in cancer precision medicine. Trends Cancer 7, 359–372. 10.1016/j.trecan.2021.01.007 33563578 PMC7969443

[B69] Nguyen H. C. T. Baik B. Yoon S. Park T. Nam D. (2023). Benchmarking integration of single-cell differential expression. Nat. Commun. 14, 1570. 10.1038/s41467-023-37126-3 36944632 PMC10030080

[B70] Nofech-Mozes I. Soave D. Awadalla P. Abelson S. (2023). Pan-cancer classification of single cells in the tumour microenvironment. Nat. Commun. 14, 1615. 10.1038/s41467-023-37353-8 36959212 PMC10036554

[B71] Overbey E. G. Das S. Cope H. Madrigal P. Andrusivova Z. Frapard S. (2022). Challenges and considerations for single-cell and spatially resolved transcriptomics sample collection during spaceflight. Cell Rep. Methods 2, 100325. 10.1016/j.crmeth.2022.100325 36452864 PMC9701605

[B72] Pan X. Li H. Zhang X. (2022). TedSim: temporal dynamics simulation of single-cell RNA sequencing data and cell division history. Nucleic Acids Res. 50, 4272–4288. 10.1093/nar/gkac235 35412632 PMC9071466

[B73] Penaranda C. Hung D. T. (2019). Single-Cell RNA sequencing to understand host–pathogen interactions. ACS Infect. Dis. 5, 336–344. 10.1021/acsinfecdis.8b00369 30702856

[B74] Peneder P. Stütz A. M. Surdez D. Krumbholz M. Semper S. Chicard M. (2021). Multimodal analysis of cell-free DNA whole-genome sequencing for pediatric cancers with low mutational burden. Nat. Commun. 12, 3230. 10.1038/s41467-021-23445-w 34050156 PMC8163828

[B75] Pensold D. Zimmer-Bensch G. (2020). “Methods for single-cell isolation and preparation,” in Advances in experimental medicine and biology. Editors Yu B. Zhang J. Zeng Y. Li L. Wang X. (Singapore: Springer Singapore), 7–27. 10.1007/978-981-15-4494-1_2 32949387

[B76] Pe’er D. Ogawa S. Elhanani O. Keren L. Oliver T. G. Wedge D. (2021). Tumor heterogeneity. Cancer Cell 39, 1015–1017. 10.1016/j.ccell.2021.07.009 34375606

[B77] Poduri A. Evrony G. D. Cai X. Elhosary P. C. Beroukhim R. Lehtinen M. K. (2012). Somatic activation of AKT3 causes hemispheric developmental brain malformations. Neuron 74, 41–48. 10.1016/j.neuron.2012.03.010 22500628 PMC3460551

[B78] Proietto M. Crippa M. Damiani C. Pasquale V. Sacco E. Vanoni M. (2023). Tumor heterogeneity: preclinical models, emerging technologies, and future applications. Front. Oncol. 13, 1164535. 10.3389/fonc.2023.1164535 37188201 PMC10175698

[B79] Proserpio V. Duval C. Falvo V. Donati G. Oliviero S. (2022). “Single-Cell sequencing for everybody,” in *Immune Receptors*. Methods in molecular biology. Editors Rast J. Buckley K. (New York, NY: Springer US), 217–229. 10.1007/978-1-0716-1944-5_15 34870822

[B80] Ren Y. Li R. Feng H. Xie J. Gao L. Chu S. (2022). Single-cell sequencing reveals effects of chemotherapy on the immune landscape and TCR/BCR clonal expansion in a relapsed ovarian cancer patient. Front. Immunol. 13, 985187. 10.3389/fimmu.2022.985187 36248860 PMC9555851

[B81] Resta R. Biesecker B. B. Bennett R. L. Blum S. Estabrooks Hahn S. Strecker M. N. (2006). A new definition of genetic counseling: National Society of Genetic Counselors’ Task Force Report. J. Gene. Counseling 15, 77–83. 10.1007/s10897-005-9014-3 16761103

[B82] Robinson T. M. Bowman R. L. Persaud S. Liu Y. Neigenfind R. Gao Q. (2023). Single-cell genotypic and phenotypic analysis of measurable residual disease in acute myeloid leukemia. Sci. Adv. 9, eadg0488. 10.1126/sciadv.adg0488 37729414 PMC10881057

[B83] Sadida H. Q. Abdulla A. Marzooqi S. A. Hashem S. Macha M. A. Akil A. S. A.-S. (2024). Epigenetic modifications: key players in cancer heterogeneity and drug resistance. Transl. Oncol. 39, 101821. 10.1016/j.tranon.2023.101821 37931371 PMC10654239

[B84] See P. Lum J. Chen J. Ginhoux F. (2018). A single-cell sequencing guide for immunologists. Front. Immunol. 9, 2425. 10.3389/fimmu.2018.02425 30405621 PMC6205970

[B85] Sharma A. K. Dhasmana N. Dubey N. Kumar N. Gangwal A. Gupta M. (2017). Bacterial virulence factors: secreted for survival. Indian J. Microbiol. 57, 1–10. 10.1007/s12088-016-0625-1 28148975 PMC5243249

[B86] Sharma S. Gioia L. Abe B. Holt M. Costanzo A. Kain L. (2018). Using single cell analysis for translational studies in immune mediated diseases: opportunities and challenges. Mol. Immunol. 103, 191–199. 10.1016/j.molimm.2018.09.020 30300798 PMC6258053

[B87] Shen X. Dai J. Guo L. Liu Z. Yang L. Gu D. (2024). Single-cell low-pass whole genome sequencing accurately detects circulating tumor cells for liquid biopsy-based multi-cancer diagnosis. npj Precis. Onc. 8, 30. 10.1038/s41698-024-00520-1 38321112 PMC10847465

[B88] Sreenivasan V. K. A. Henck J. Spielmann M. (2022). Single-cell sequencing: promises and challenges for human genetics. Med. Genet. 34, 261–273. 10.1515/medgen-2022-2156 38836091 PMC11006387

[B89] Stuart T. Butler A. Hoffman P. Hafemeister C. Papalexi E. Mauck W. M. (2019). Comprehensive integration of single-cell data. Cell 177, 1888–1902. 10.1016/j.cell.2019.05.031 31178118 PMC6687398

[B90] Sulen A. Islam S. Wolff A. S. B. Oftedal B. E. (2020). The prospects of single‐cell analysis in autoimmunity. Scand. J. Immunol. 92, e12964. 10.1111/sji.12964 32869859

[B91] Sultana A. Alam M. S. Liu X. Sharma R. Singla R. K. Gundamaraju R. (2023). Single-cell RNA-seq analysis to identify potential biomarkers for diagnosis, and prognosis of non-small cell lung cancer by using comprehensive bioinformatics approaches. Transl. Oncol. 27, 101571. 10.1016/j.tranon.2022.101571 36401966 PMC9676382

[B92] Tang X. Huang Y. Lei J. Luo H. Zhu X. (2019). The single-cell sequencing: new developments and medical applications. Cell Biosci. 9, 53. 10.1186/s13578-019-0314-y 31391919 PMC6595701

[B93] Tang Q. Li W. Huang J. Wu Y. Ma C. Tu Y. (2023). Single-cell sequencing analysis of peripheral blood in patients with moyamoya disease. Orphanet J. Rare Dis. 18, 174. 10.1186/s13023-023-02781-8 37400835 PMC10318666

[B94] Thorpe J. Osei-Owusu I. A. Avigdor B. E. Tupler R. Pevsner J. (2020). Mosaicism in human health and disease. Annu. Rev. Genet. 54, 487–510. 10.1146/annurev-genet-041720-093403 32916079 PMC8483770

[B95] Towle-Miller L. M. Miecznikowski J. C. (2022). MOSCATO: a supervised approach for analyzing multi-omic single-cell data. BMC Genomics 23, 557. 10.1186/s12864-022-08759-3 35927608 PMC9351124

[B96] Vallejos C. A. Risso D. Scialdone A. Dudoit S. Marioni J. C. (2017). Normalizing single-cell RNA sequencing data: challenges and opportunities. Nat. Methods 14, 565–571. 10.1038/nmeth.4292 28504683 PMC5549838

[B97] Velmeshev D. Perez Y. Yan Z. Valencia J. E. Castaneda-Castellanos D. R. Wang L. (2023). Single-cell analysis of prenatal and postnatal human cortical development. Science 382, eadf0834. 10.1126/science.adf0834 37824647 PMC11005279

[B98] Wajda A. Sivitskaya L. Paradowska-Gorycka A. (2021). Application of NGS technology in understanding the pathology of autoimmune diseases. J. Clin. Med. 10, 3334. 10.3390/jcm10153334 34362117 PMC8348854

[B99] Walker C. R. Li X. Chakravarthy M. Lounsbery-Scaife W. Choi Y. A. Singh R. (2024). Private information leakage from single-cell count matrices. Cell 187, 6537–6549.e10. 10.1016/j.cell.2024.09.012 39362221 PMC11568916

[B100] Wang J. Zou Q. Lin C. (2022). A comparison of deep learning-based pre-processing and clustering approaches for single-cell RNA sequencing data. Briefings Bioinforma. 23, bbab345. 10.1093/bib/bbab345 34472590

[B101] Wen L. Tang F. (2022). Recent advances in single-cell sequencing technologies. Precis. Clin. Med. 5, pbac002. 10.1093/pcmedi/pbac002 35821681 PMC9267251

[B102] Wiedmeier J. E. Noel P. Lin W. Von Hoff D. D. Han H. (2019). “Single-Cell sequencing in precision medicine,” in *Precision medicine in cancer therapy*. Cancer treatment and research. Editors Von Hoff D. D. Han H. (Cham: Springer International Publishing), 237–252. 10.1007/978-3-030-16391-4_9 31209848

[B103] Wu A. R. Neff N. F. Kalisky T. Dalerba P. Treutlein B. Rothenberg M. E. (2014). Quantitative assessment of single-cell RNA-sequencing methods. Nat. Methods 11, 41–46. 10.1038/nmeth.2694 24141493 PMC4022966

[B104] Xu J. Liao K. Yang X. Wu C. Wu W. (2021). Using single-cell sequencing technology to detect circulating tumor cells in solid tumors. Mol. Cancer 20, 104. 10.1186/s12943-021-01392-w 34412644 PMC8375060

[B105] Xu Y. Wang Y. Liang L. Song N. (2022). Single-cell RNA sequencing analysis to explore immune cell heterogeneity and novel biomarkers for the prognosis of lung adenocarcinoma. Front. Genet. 13, 975542. 10.3389/fgene.2022.975542 36147484 PMC9486955

[B106] Yadav S. Mehta P. Soni J. Chattopadhyay P. Devi P. Habyarimana T. (2023). Single-cell RNA-Seq reveals intracellular microbial diversity within immune cells during SARS-CoV-2 infection and recovery. iScience 26, 108357. 10.1016/j.isci.2023.108357 38026191 PMC10663746

[B107] Yang Y. Li H. Liu P. Jia J. Wei L. Chen X. (2025). Multitemporal single-cell profiling uncovers alveolar IL1βhi neutrophils: a significant indicator of CARDS progression. Clin. Transl. Med. 15, e70479. 10.1002/ctm2.70479 40999573 PMC12463734

[B108] Yip S. H. Wang P. Kocher J.-P. A. Sham P. C. Wang J. (2017). Linnorm: improved statistical analysis for single cell RNA-seq expression data. Nucleic Acids Res. 45, e179. 10.1093/nar/gkx828 28981748 PMC5727406

[B109] Yu X. Abbas-Aghababazadeh F. Chen Y. A. Fridley B. L. (2021). “Statistical and bioinformatics analysis of data from bulk and single-cell RNA sequencing experiments,” in *Translational bioinformatics for therapeutic development*. Methods in molecular biology. Editor Markowitz J. (New York, NY: Springer US), 143–175. 10.1007/978-1-0716-0849-4_9 PMC7771369 32926366

[B110] Zeb Q. Wang C. Shafiq S. Liu L. (2019). “An overview of single-cell isolation techniques,” in Single-Cell omics. Elsevier, 101–135. 10.1016/B978-0-12-814919-5.00006-3

[B111] Zhang M. Liu S. Miao Z. Han F. Gottardo R. Sun W. (2022a). IDEAS: individual level differential expression analysis for single-cell RNA-seq data. Genome Biol. 23, 33. 10.1186/s13059-022-02605-1 35073995 PMC8784862

[B112] Zhang A. Miao K. Sun H. Deng C.-X. (2022b). Tumor heterogeneity reshapes the tumor microenvironment to influence drug resistance. Int. J. Biol. Sci. 18, 3019–3033. 10.7150/ijbs.72534 35541919 PMC9066118

[B113] Zhang K. Chen Y. Zhu J. Ge X. Wu J. Xu P. (2023). Advancement of single-cell sequencing for clinical diagnosis and treatment of pancreatic cancer. Front. Med. 10, 1213136. 10.3389/fmed.2023.1213136 37720505 PMC10501729

[B114] Zhang K. K. Li J. Jeon M. Ramos K. S. (2024). “Single-Cell mRNA sequencing in precision medicine: promise and challenges,” in Comprehensive precision medicine. Elsevier, 59–72. 10.1016/B978-0-12-824010-6.00028-9

[B115] Zhao M. Jiang J. Zhao M. Chang C. Wu H. Lu Q. (2021). The application of single-cell RNA sequencing in studies of autoimmune diseases: a comprehensive review. Clin. Rev. Allerg. Immunol. 60, 68–86. 10.1007/s12016-020-08813-6 33236283

[B116] Zhao M. Wang C. Li P. Sun T. Wang J. Zhang S. (2023). Single-cell RNA sequencing reveals the transcriptomic characteristics of peripheral blood mononuclear cells in hepatitis B vaccine non-responders. Front. Immunol. 14, 1091237. 10.3389/fimmu.2023.1091237 37593735 PMC10431960

[B117] Zhu C.-X. Qin J.-J. (2023). Single-cell RNA sequencing and spatial transcriptomic technologies and applications in exploring gastric cancer: a review. Oncol. Adv. 1, 6–16. 10.14218/OnA.2023.00039

[B118] Zou D. Qi J. Wu W. Xu D. Tu Y. Liu T. (2021a). Applications of single-cell sequencing in dermatology. Med. Sci. Monit. 27, e931862. 10.12659/MSM.931862 34011922 PMC8147034

[B119] Zou A. Ramanathan S. Dale R. C. Brilot F. (2021b). Single-cell approaches to investigate B cells and antibodies in autoimmune neurological disorders. Cell Mol. Immunol. 18, 294–306. 10.1038/s41423-020-0510-z 32728203 PMC8027387

